# A multiple criteria decision analysis based approach to remove uncertainty in SMP models

**DOI:** 10.1038/s41598-022-27059-0

**Published:** 2022-12-26

**Authors:** Gokul Yenduri, Thippa Reddy Gadekallu

**Affiliations:** School of Information Technology and Engineering, Vellore Institute of Technology, Vellore, Tamil Nadu 632014 India

**Keywords:** Engineering, Mathematics and computing

## Abstract

Software has to be updated frequently to match the customer needs. If software maintainability is not given priority, it affects the software development life cycle and maintenance expenses, which deplete organizational assets. Before releasing software, maintainability must be estimated, as the impact of bugs and errors can affect the cost and reputation of the organization after deployment. Regardless of the programming paradigm, it’s important to assess software maintainability. Many software maintainability prediction models’ compatibilities with new programming paradigms are criticized because their limited applicability over heterogeneous datasets. Due this challenge small and medium-sized organizations may even skip the maintainability assessment, resulting in huge lose to such organizations. Motivated by this fact, we used Genetic Algorithm optimized Random Forest technique (GA) for software maintainability prediction models over heterogeneous datasets. To find optimal model for software maintainability prediction, the Technique for Order preference by Similarity to Ideal Solution (TOPSIS), a popular multiple-criteria decision-making model, is adopted. From the results, it is concluded that the GA is optimal for predicting maintainability of software developed in various paradigms.

## Introduction

In today’s world, innovation is taking place rapidly, and as a result, the world is moving toward complete automation with the help of AI. The combination of AI and automation is very fascinating and covers so many problems, like deliberate action, planning, understanding open environments, and interacting with other robots^1^. The AI and automation duo have had a lot of impact on many fields, like employment, processing mathematical problems, the hospitality industry, smart cities, and many others^2^. Robotics has always proved productive for AI research. This combination also increases the capabilities of robotics in assisting humans at factories, hospitals, farms, etc.^3^. As a result, there has been a significant increase in the software sector, and clients expect high-quality software, which has become a challenge for software developers^4^. Companies are competing to bring the best-automated products to market and provide a large number of upgrades to already released products to stay ahead in this competitive market^5^. According to ISO/IEC 25010^6^, maintainability is a critical factor for quality of software. As the software is highly agile, it is always evolving to meet the needs of the clients. So, the maintainability of a product should be given top priority before it is released to the market, as it directly affects the cost. The distinguishing characteristic of any software is “change”, and as a result, extra care should be taken while developing software. As a result, the software is expected to be easily modified. The maintainability of a software is dependent on internal and external quality factors.

Software maintainability prediction models can aid in minimizing the effort needed to maintain software. When software maintainability is evaluated early in the development process, organizations may more effectively allocate and manage their resources. This work used Genetic Algorithm optimized Random Forest (GA) for predicting the maintainability of software developed in various paradigm. This work also validated over NASA application datasets JM1, KC1, MC1, MW1, PC1, PC2, PC3, PC4, and PC5 along with popular commercial application datasets UIMS and QUES. To find the optimal model for software maintainability predictions three popular Multiple Criteria Decision Analysis based Approach’s (MCDAs).

The rest of the paper is structured as follows: The overview of software maintainability measures and prediction approaches used in this work is discussed in “Software maintainability measures and prediction approaches” section. Recent works are discussed in “Recent works” section. The proposed methodology for predicting software maintainability is detailed in “Predicting software maintainability” section. “Results and discussion” section describes the techniques that are used, along with the results and discussion. “Threat to validity” section discusses potential threats to the proposed work’s validity. “Conclusion” section includes the research findings and detailed conclusions.

## Software maintainability measures and prediction approaches

Software maintainability is hard to measure directly. So, previous researchers used predictive models. Measuring the software’s maintainability is dependent on the metrics used, and identifying the appropriate metrics for source code is the most difficult aspect of maintainability prediction. Researchers have estimated software maintainability using the MI, Change and other measures^7^. The MI is most admired among the researchers as it is validated by Hewlett–Packard (HP).

The MI in Eq. (1) was proposed by Coleman et al.^8^. It is a combination of several metrics, including Halstead’s Volume (HV), McCabe’s cyclomatic complexity (CC), lines of code (LOC), and percentage of comments (COM), and is validated by HP^3^.

1 $$MI=171-5.2\mathrm{ln}V-0.23G-16.2\mathrm{ln}L.$$

The Software Engineering Institute (SEI) has derived an MI in Eq. (2), which is based on the Coleman work in the year 1997. The MI ranges from 0 to 100, where 0 indicates that the software is hard to maintain and as the range increases towards 100, it indicates that the software is maintainable^9^.

2 $$MI=171-5.2{\mathrm{log}}_{2}V-0.23G-16.2{\mathrm{log}}_{2}L+50\mathrm{sin}\left(\sqrt{2.4\mathrm{C}}\right).$$

Radon^10^, a popular Python tool, uses another derivative, MI, as depicted in Eq. (3). It is calculated based on the following SEI and Visual Studio derivatives.

3 $$MI=\mathrm{max}[0,100171-5.2\mathrm{ln}V-0.23G-16.2\mathrm{ln}L+50\mathrm{sin}50\mathrm{sin}(\sqrt{2.4C}))].$$

In the year 2011, Microsoft team blog^11^ has reset the ranges or thresholds to 0–9 = Red, 10–19 = Yellow and 20–100 = Green which indicates poor, medium, and high range of maintainability with minor modifications as shown below in Eq. (4).

4 $$MI=\frac{ MAX(0,(171 - 5.2 \times \mathrm{ln}(HV) - 0.23 \times (CC) - 16.2 \times \mathrm{ln}(LOC))\times 100)}{171}.$$

Li and Henry found that complexity, coupling, cohesion, and inheritance metrics had a substantial link with class change volumes after examining two projects, UIMS (User Interface Management System) and QUES (Quality Evaluation System). With the aid of Classic-Ada, information on maintenance activities was collected from two commercial systems. The data was collected over 3 years. The number of lines per class that have been edited determines the maintenance effort. According to Eq. (5), the metric Change might include adding or removing code lines. This suggested measure can be employed to assess an object-oriented system's maintainability.

5 $$\mathrm{CHANGE}=\mathrm{F}\left(WMC,DIT,NOC,RFC,LCOM,MPC,DAC,NOM,S1,S2\right).$$

However, no commonly accepted measure for identifying the relevant code metrics or prediction models is available^12^.

Researchers have employed individual prediction models like SVR, ANN, LSTM and ensemble models to improve the accuracy prediction of individual models^13^. They can be classified into two major types: homogenous that uses the same type of individual models, and heterogenous that uses different types of individual models. There is a scarcity of the data that is needed to analyze cross-domain projects and other benchmark datasets.

## Recent works

In this section, we presented a detailed overview of recent works in predicting the maintainability of software.

In 2021, Iqbal et al.^13^ used a supervised learning approach to identify the changes that were required in the legacy system’s current software components. New requirements and defect kinds necessitated a thorough redesign of the software components’ interfaces. The software maintainability was assessed using the naive Bayes classifier, a machine learning technique. Software components designed with the inverse criteria in mind were found to be error-free and easily adaptable to client needs. The authors employed limited datasets to estimate maintainability using only one machine learning technique, which is a significant flaw in this work. It can be encapsulated using heterogeneous software and recent algorithms.

In 2021, Lakra et al.^14^ applied hyperparameter tuning on five regression-based ML algorithms like random forest, ridge regression, support vector regression, stochastic gradient descent, and gaussian process regression for two commercial object-oriented datasets, namely QUES and UIMS. The results exhibited substantial improvements when compared to the existing base models. The work primarily focuses only on fine-tuning models, lacks the usage of heterogeneous software, and has the limitation of not addressing the uncertainty in software maintainability prediction models. The work also uses only a few datasets, which is a serious drawback.

In 2020, Elmidaoui et al.^15^ conducted a study on empirical evidence for the accuracy of software product maintainability prediction (SPMP) using ML techniques. The after-effects of about 77 studies that were published between 2000 and 2018 are inspected in this study based on the following criteria: maintainability prediction approaches, validation methods, accuracy criteria, the overall accuracy of ML techniques, and the techniques with the best performance. In the maximum number of studies, ML techniques’ performance exceeded the non-ML techniques’, such as regression analysis (RA), whereas fuzzy and neuro-fuzzy (FNF) outscored SVM/R, DT, and ANN. When several techniques were claimed to be superior, no specific technique could be recognized as the best, which is a serious limitation in this work.

In 2020, Malhotra et al.^16^ used nine oversampling and three under-sampling approaches on unbalanced data. A detailed comparison of fourteen ML and fourteen search-based strategies has been taken into consideration to predict the class maintainability. This work supports the use of the Safe-Level Synthetic Minority Oversampling Technique (Safe-SMOTE) in handling imbalanced data when predicting class maintainability. This work has certain limitations compared to heterogeneous techniques and recent ML techniques. This work has an ambiguity in choosing an appropriate algorithm for estimating maintainability, which is a major drawback.

In 2020, Gupta et al.^17^ proposed an enhanced-RFA (Random Forest Algorithm) technique for software maintainability prediction. The suggested method combines the random forest (RF) algorithm with three widely used feature selection techniques: chi-square, RF, and linear correlation filter, as well as a re-sampling strategy to increase the core RF algorithm’s prediction accuracy. Using R^2^, the performance of enhanced-RFA is assessed on two commercially available datasets, namely QUES and UIMS. The proposed approach performs much better than RFA for the specified datasets using chi-square, RF, and linear correlation filter approaches. This work has limitations in comparison to heterogeneous techniques and does not compare itself to recent ML techniques.

In 2020, Malhotra et al.^18^ implemented several ML, statistical (ST) and hybridization (HB) techniques to create prediction models for software maintainability in this work. The important finding is that ML-based models outperform ST models in terms of overall performance. The use of HB methods for software maintainability prediction is restricted. It is encouraging that this work has reported the prediction performance of a few models developed using HB techniques, but no conclusive results about the performance of any of these techniques are reported and this paper ignores the metaheuristics.

From the above literature survey, it is understood that the most popular SMP techniques are the statistical model and individual ML models. In SMP, the ML models performed better than the statistical models. To increase the effectiveness of the models and their accuracy, the researchers are employed ensemble models. Several SMP models claim superiority. However, their performance across diverse programming paradigms is questionable, making the selection of an SMP model uncertain. This necessitates an approach focused on minimization of effort when selecting an SMP model for diverse programming paradigms.

The summary of the recent works is depicted in Table 1.

**Table 1:** Recent works on software maintainability prediction.

References	Year	Models used	Best models	Limitations
^13^	2021	Machine learning technique	Naive Bayes classifier	Limited datasets
^14^	2021	Machine learning technique with hyper parameter tuning	Grid search method	Uncertainty in software maintainability prediction models
^15^	2020	Machine learning techniques and statistical techniques	ML techniques	Uncertainty in software maintainability prediction models
^16^	2020	Machine learning techniques and search based (SB) techniques	Safe-SMOTE with ML technique	Limitations over heterogeneous techniques and recent ML techniques
^17^	2020	RF algorithm with re-sampling technique	RF algorithm with re-sampling technique	Limits over heterogeneous techniques and did not compare itself with recent ML techniques
^18^	2020	Machine learning techniques, Statistical (ST), and Hybridized (HB) techniques	Machine learning (ML)	No conclusive results about the performance of any of these techniques is reported

## Predicting software maintainability

This section presents a overview of proposed methodology for assessing the maintainability of automated software.

### Experimental setup

The software maintainability prediction models are implemented using R Programming. The aim is to reduce the prediction error and improve the robustness of the proposed model. GA compares its performance over other models like step-wise regression, support vector machine, NN, MARS, and CART. The datasets are obtained from the Li and Henry’s work and PROMISE repository. Quality Evaluation System (QUES) and User Interface System (UIMS) datasets of commercial software products were originally released by Li and Henry in 1993 in their work on object-oriented metrics that predict maintainability^19–22^. QUES has 71 classes whereas UMIS contains 39 classes. CM1, JM1, KC1, KC3, MC1, MC2, MW1, PC1, PC2, PC3, PC4, and PC5 were made available through NASA Metrics Data Program by Tim Menzies in year 2004 and are available in PROMISE repository^23^. The details of these datasets are described in Table 2.

**Table 2:** Datasets used in this research.

Dataset	Language	Feature	Instances	Source
UIMS	JAVA	11	39	Li and Henry^19–22,24^
QUES	JAVA	11	71	Li and Henry^19–22,24^
CM1	C	40	505	Promise^25^
JM1	C	21	10,878	Promise^25^
KC1	C++	21	2107	Promise^25^
KC3	JAVA	40	458	Promise^25^
MC1	C&C++	39	9466	Promise^25^
MC2	C	40	161	Promise^25^
MW1	C	40	403	Promise^25^
PC1	C	40	1107	Promise^25^
PC2	C	40	5589	Promise^25^
PC3	C	40	1563	Promise^25^
PC4	C	40	1458	Promise^25^
PC5	C++	39	17,186	Promise^25^

**Figure 1:**
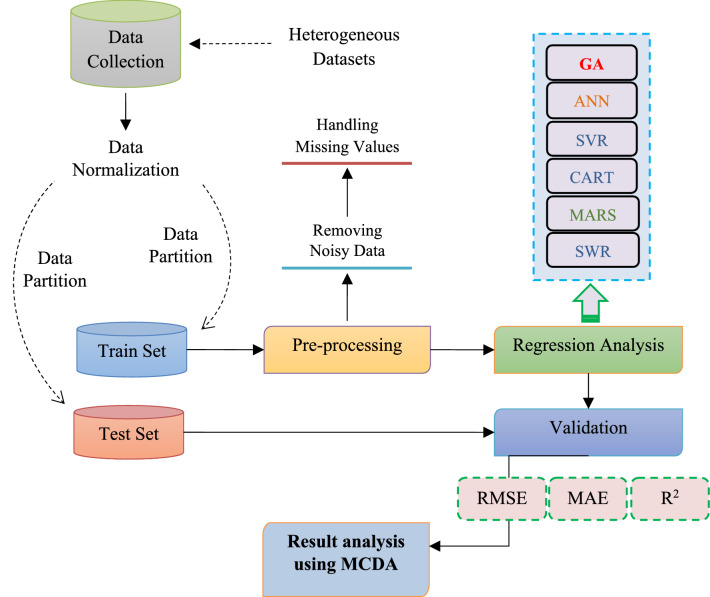
A methodology for assessing the maintainability of automated software.

In contrast, the distinction is drawn in correspondence with the comparative analysis R^2^, MAE and RMSE as shown in Fig. 1.

### Objective function

The motivation of this paper is to find the better software maintainability prediction model with less error ratio when applied to heterogeneous datasets. The error is estimated based on the difference between $MI^{actual}$ and the predicted $MI^{predicted}$, which is defined in Eq. (6).

6 $$ Error = \min \left( {MI^{actual} - MI^{predicted} } \right). $$

### Solution encoding

**Figure 2:**
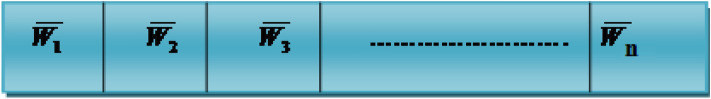
Solution encoding.

$\overline{W}$ that represents the optimized weight, and the solution proposed is illustrated in Fig. 2.

### Genetic algorithm

**Figure 3:**
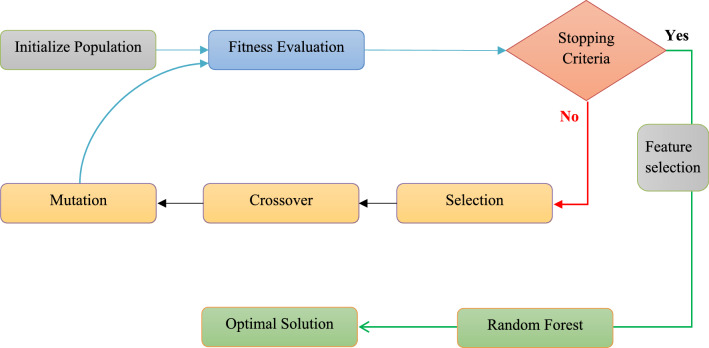
Genetic algorithm optimized random forest algorithm.


To improve prediction, this paper uses an evolutionary computing and most admired algorithm, GA^26^. GA algorithm imitates human evolution, in particular, gene evolution, and is inspired by Charles Darwin’s theory of natural evolution. This algorithm represents the natural selection mechanism where the fittest individuals are chosen for succession to generate next-generation offspring. Parallelism is supported by the genetic algorithm, which is easily modified and adaptable to various problems. It is easy to disseminate and can search a large and diverse solution space. A non-knowledge-based optimization process is used to evaluate the fitness function. Finding the global optimum and avoiding becoming trapped in the local optimum is simple^27^. A suite of potential solutions can be returned by multi-objective optimization. GA is appropriate for large-scale and diverse optimization problems. The five rules of applying genetic algorithm are shown in Fig. 3.

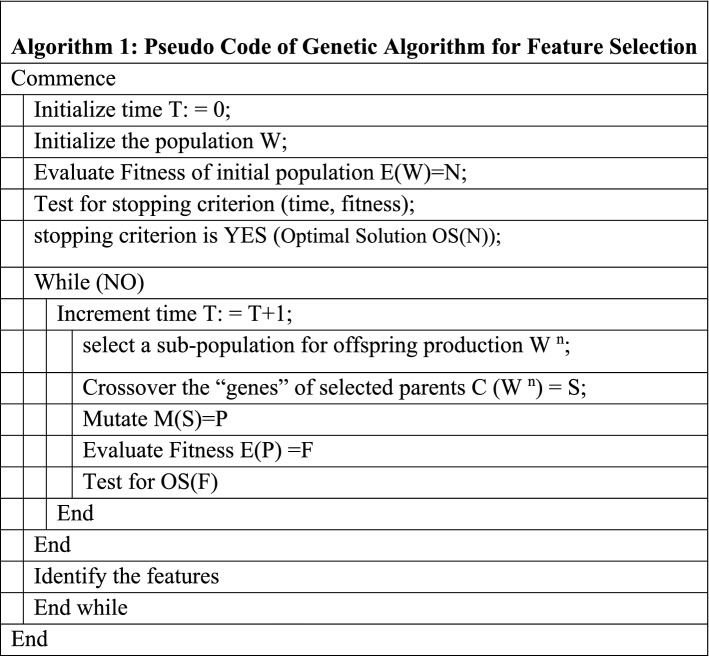


While comparing GA with other popular algorithms, GA can deal with multi-model problems more efficiently. Further, it uses a good initial solution to start its iteration process. Simultaneously, the fitness of the whole population is evaluated and multiple individuals are stochastically selected from the current based on the fitness value then the new population is formed where the process iterates until the best solution is reached.

## Results and discussion

The proposed methodology for software maintainability prediction is implemented using R programming. The developed model aims to reduce the error rate. Six popular techniques, namely, SWR, SVM, NN, MARS, GA, and CART are considered for software maintainability prediction and their performance is evaluated based on RMSE, MAE, and R^2^.

### Performance analysis

A critical step in any empirical study is determining the predicted model’s accuracy. The model predicts the value of the dependent variable, which is then compared to the actual value to discover errors. The current work compares various popular ML techniques, statistical (ST), and metaheuristic techniques using the following measures.

7 $$MAE=1/N\sum \limits_{i=1}^{N} \frac{Actual \, value-Anticipated \, value}{Actual \, value} .$$

The mean absolute error (MAE)^8^ in Eq. (7), is a standardized measure used to find differences between the actual and anticipated values of a dependent variable. MAE calculates the difference between the actual and anticipated values first and then divides the result by the actual value. After that, each data point’s absolute value is added together and divided by the entire number of data points.

8 $$RMSE=(\sqrt{1/n\sum (Actual \, value-Predicted \, value)2}.$$

The difference between predicted and actual values for each class is squared, then averaged, and finally the square root of the average value is calculated in RMSE^8^ as Eq. (8).

In a regression model, R^2^^8^ Eq. (9), is the amount of variation explained by an independent variable or factors for a dependent variable. The R^2^ value indicates how much the variance of one variable explains the variance of the other.

9 $$\text{R squared }= \text{ 1 } - \, \frac{\text{First sum of errors}}{\text{Second sum of errors}}.$$

In regression analysis, the residual squared error (R^2^), mean absolute error (MAE), and root mean squared error (RMSE) metrics are used to assess the model’s performance. The lower the value of MAE, RSQ, and RMSE, the more accurate a regression model is considered to be. Compared to the MSE, the mean squared error (MSE) and RMSE penalize the big prediction errors (MAE). However, because it has the same units as the dependent variable, RMSE is more commonly used to evaluate the efficiency of the regression model when compared to other random models than MSE (Y-axis).

The UIMS dataset is private and the source code is entirely developed using Java programming which contains 39 instances and 11 features. The genetic algorithm has achieved better performance compared to other models.

The QUES dataset is private and the source code is entirely developed using Java programming which contains 71 instances and 11 features. Neural networks have achieved better performance compared to other models.

The CM1 dataset is public and the source code is entirely developed using C programming which contains 505 instances and 40 features. Neural networks have achieved better performance compared to other models.

The JM1 dataset is public and the source code is entirely developed using C programming which contains 10,878 instances and 21 features. Neural networks^26^ have achieved better performance compared to other models.

The KC1 dataset is public and the source code is entirely developed using C++ programming which contains 2107 instances and 21 features. Genetic algorithm has achieved better performance compared to other models.

The KC3 dataset public and the source code is entirely developed using Java programming which contains 458 instances and 40 features. MARS have achieved better performance compared to other models.

The MC1 dataset is public and the source code is entirely developed using C and C++ programming which contains 9466 instances and 39 features. Genetic algorithm has achieved better performance compared to other models.

The MC2 dataset is public and the source code is entirely developed using C programming which contains 161 instances and 40 features. MARS has achieved better performance compared to other models.

The MW1 dataset is public and the source code is entirely developed using C programming which contains 403 instances and 40 features. Genetic algorithm has achieved better performance compared to other models.

The PC1 dataset is public and the source code is entirely developed using C programming which contains 1107 instances and 40 features. MARS has achieved better performance compared to other models.

The PC2 dataset is public and the source code is entirely developed using C programming which contains 5589 instances and 40 features. Genetic algorithm has achieved better performance compared to other models.

The PC3 dataset is public and the source code is entirely developed using C programming which contains 1563 instances and 40 features. MARS has achieved better performance compared to other models.

The PC3 dataset is public and the source code is entirely developed using C programming which contains 1458 instances and 40 features. Genetic algorithm has achieved better performance compared to other models.

The PC5 dataset is public and the source code is entirely developed using C programming which contains 17,186 instances and 39 features. Genetic algorithm has achieved better performance compared to other models.

**Figure 4:**
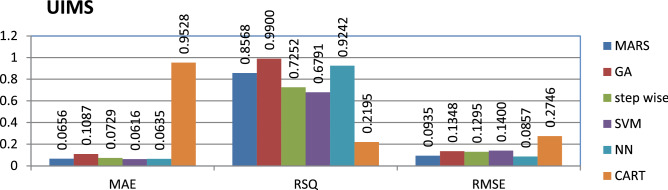
Analysis on renowned maintainability prediction models using evaluation measures on UIMS dataset.

**Figure 5:**
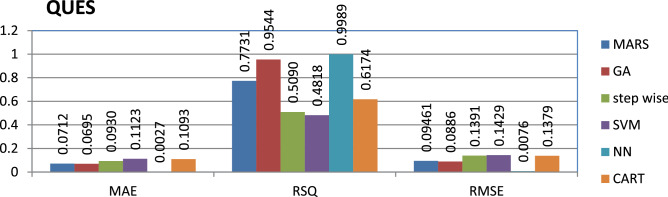
Analysis on renowned maintainability prediction models using evaluation measures on QUES dataset.

**Figure 6:**
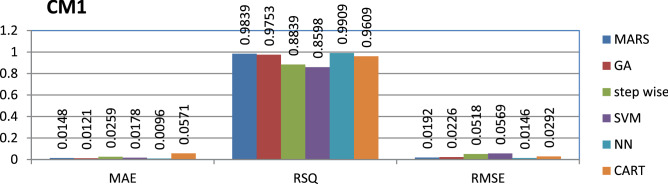
Analysis on renowned maintainability prediction models using evaluation measures on CM1 dataset.

**Figure 7:**
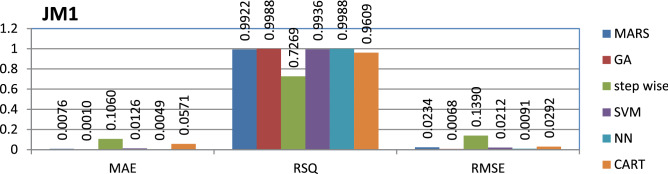
Analysis on renowned maintainability prediction models using evaluation measures on JM1 dataset.

**Figure 8:**
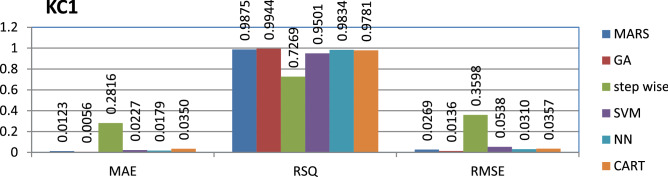
Analysis on renowned maintainability prediction models using evaluation measures on KC1 dataset.

**Figure 9:**
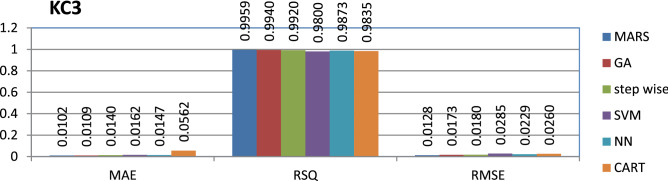
Analysis on renowned maintainability prediction models using evaluation measures on KC3dataset.

**Figure 10:**
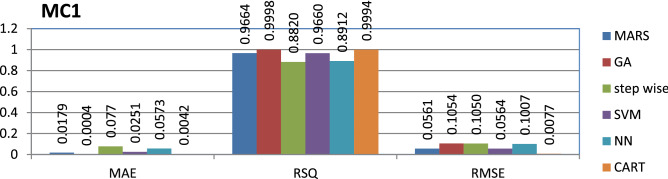
Analysis on renowned maintainability prediction models using evaluation measures on MC1 dataset.

**Figure 11:**
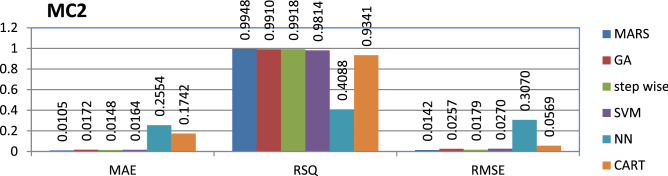
Analysis on renowned maintainability prediction models using evaluation measures on MC2 dataset.

**Figure 12:**
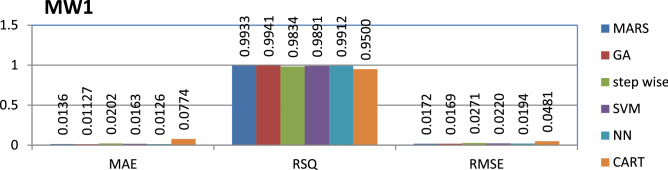
Analysis on renowned maintainability prediction models using evaluation measures on MW1 dataset.

**Figure 13:**
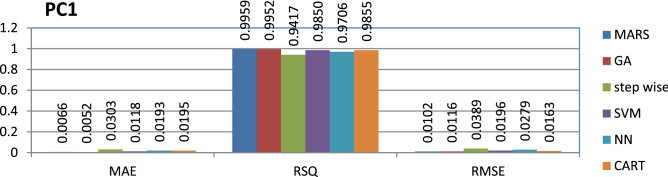
Analysis on renowned maintainability prediction models using evaluation measures on PC1 dataset.

**Figure 14:**
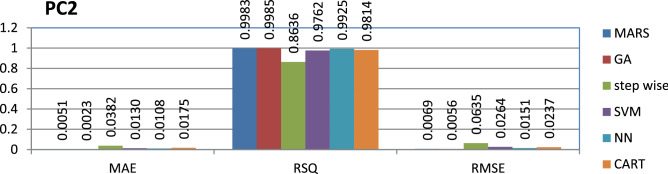
Analysis on renowned maintainability prediction models using evaluation measures on PC2 dataset.

**Figure 15:**
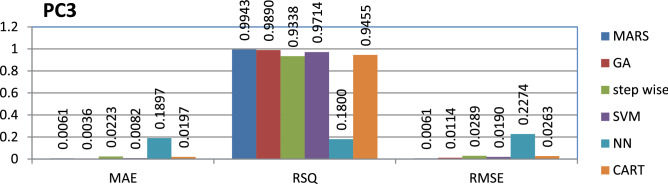
Analysis on renowned maintainability prediction models using evaluation measures on PC3 dataset.

**Figure 16:**
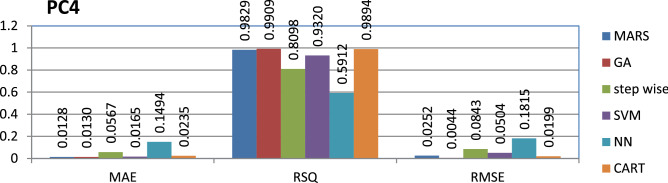
Analysis on renowned maintainability prediction models using evaluation measures on PC4 dataset.

**Figure 17:**
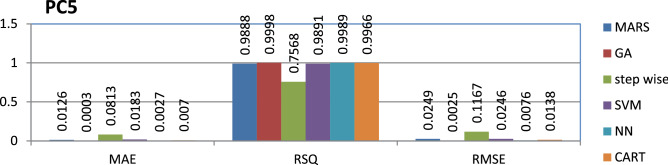
Analysis on renowned maintainability prediction models using evaluation measures on PC5 dataset.

The results of all the performance measures (MAE, RSQ, and RMSE) on various datasets are presented in Figs. 4, 5, 6, 7, 8, 9, 10, 11, 12, 13, 14, 15, 16 and 17. Based on UIMS, QUES, CM1, and JM1 data sets the best algorithm is NN for predicting the maintainability, (RMSE(UIMS) = 8.57%, RMSE(QUES) = 0.7%, RMSE(CM1) = 1.46%, RMSE(JM1) = 0.91%). The best algorithm for the datasets KC1, MW1, PC2, PC4, PC5, is GA^28^ with (RMSE(KC1) = 0.13%, RMSE(MW1) = 1.6%, RMSE(PC2) = 0.5%, RMSE(PC4) = 0.4%, RMSE(PC5) = 0.2%). MARS algorithm is the best one for predicting the maintainability for the datasets KC3, MC2, PC1, and PC3, bearing the (RMSE(KC3) = 1.2%, RMSE(MC2) = 1.4%, RMSE(PC1) = 1.0%, RMSE(PC3) = 0.6%). Finally, the best algorithm for predicting the maintainability of the dataset MC1 is CART (RMSE (MC1) = 0.77%). These results reveal that GA is the best algorithm for 36% of the data sets, followed by NN^29^, MARS (29%). The worst algorithms for predicting maintainability are CART (1%), stepwise, and SVM.

The results show that the best models in SMP are GA, NN, and MARS. These SMP models outperformed other models on heterogeneous datasets. The GA algorithm also outperformed other algorithms on datasets with a large number of instances and features. It is interesting to note that the performance of GA has decreased with some datasets that have a large number of features but a small number of instances. It is understood that GA works best when the search space is large and there are a large number of parameters involved. However, the applicability of GA over all the heterogeneous datasets is still questionable^30^ and still creates uncertainty in SMP model selection. To overcome this issue, we used a MCDA approach to prove its superiority of GA over all the heterogeneous datasets.

### Technique for order preference by similarity to ideal solution (TOPSIS)

MCDA model, the TOPSIS^28^, was applied for ranking algorithms on different datasets. It computes the distances between a real solution and its ideal as well as negative ideal counterparts^31^. We make use of the following algorithm.

*Step 1* Determine the normalized

10 $${r}_{ij}=\frac{{\chi }_{ij}}{\sqrt{{\sum }_{j=1}^{J}{\chi }_{ij}^{2}}}.$$

In Eq. (10), j = 1, 2…, J and I = 1, 2…, n represent the feature selection indices, respectively.

*Step 2* Calculate weighted

11 $${v}_{ij}={w}_{i}{r}_{ij}.$$

In Eq. (11), ${\mathrm{w}}_{\mathrm{i}}$ represents the weight.

*Step 3* Find the ideal solution ${\mathrm{S}}^{+}$ and the negative ideal solution ${\mathrm{S}}^{-}$

12 $${S}^{+}=\left\{{v}_{1}^{+}, , {v}_{n}^{+}\right\}=\left(\mathrm{ max }{v}_{ij}|ij\in {I}^{\mathrm{^{\prime}}}\right), \left(\mathrm{min}{v}_{ij}|i\in {I}^{\mathrm{^{\prime}}\mathrm{^{\prime}}}\right),$$

13 $${S}^{-}=\left\{{v}_{1}^{-}, , {v}_{\mathfrak{n}}^{-}\right\}=\left(\mathrm{ min }{v}_{ij}|i\in {I}^{^{\prime}}\right), \left(\mathrm{ max }{v}_{ii}|i\in {I}^{\mathrm{^{\prime}}\mathrm{^{\prime}}}\right).$$

In Eqs. (12) and (13), I′ and I′′ represent the benefit criteria and cost criteria, respectively. Stability, TPR, TNR, accuracy and AUC are benefit criteria. Runway.

*Step 4* Calculate the Euclidean distance between the real and ideal solutions using Eqs. (14) and (15).

14 $${D}_{j}^{+}=\sqrt{{\sum }_{i=1}^{n}{\left({v}_{ij}-{v}_{i}^{+}\right)}^{2}},$$

15 $${D}_{j}^{-}=\sqrt{{\sum }_{i=1}^{\mathrm{n}}{\left({v}_{ij}-{v}_{i}^{-}\right)}^{2}}.$$

*Step 5* Calculate ${R}_{j}^{+}$ using Eq. (16).

16 $${R}_{j}^{+}=\frac{{D}_{j}^{-}}{{D}_{j}^{+}+{D}_{j}^{-}}.$$

*Step 6* Rank feature selection methods by maximizing ${R}_{j}^{+}$ Eq. (16).

To further remove the uncertainty in SMP models, popular multi-criteria decision-making technique, the TOPSIS method, was applied for ranking algorithms on different data sets^32^. TOPSIS provides trade-offs between criteria, allowing a good performance in one criterion to offset a bad result in another. Instead of using non-compensatory approaches, which include or exclude alternate solutions based on strict cut-offs, this offers a more realistic modelling approach when compared to Multi-objective Optimization on the basis of Ratio Analysis (MOORA) and A New Additive Ratio Assessment method (ARAS).

The ranking algorithms on different data sets are presented in Table 3. From Table 3, it is observed that GA is the best model for predicting the maintainability of heterogeneous software. Further, it is observed that the correlation between TOPSIS and the other two MCDAs (MOORA and ARAS) used in this study was strong^33^. Based on the performance analysis and MCDA ranking, it is understood that GA is an optimum model for predicting the maintainability of heterogeneous software.

**Table 3:** Comparative analysis of multiple criteria decision analysis on SMP models.

Data sets	Comparative analysis of multiple criteria decision analysis on SMP models																	
	MCDM models TOPSIS–MOORA–ARAS SMP models Step-wise regression (R)–support vector machine (S)–neural networks (N)–multivariate adaptive regression splines (M)–genetic algorithm optimized random forest (G)																	
	TOPSIS	MOORA	ARAS	TOPSIS	MOORA	ARAS	TOPSIS	MOORA	ARAS	TOPSIS	MOORA	ARAS	TOPSIS	MOORA	ARAS	TOPSIS	MOORA	ARAS
	Rank 1			Rank 2			Rank 3			Rank 4			Rank 5			Rank 6		
CM1	N	N	N	M	G	G	G	M	M	S	S	S	R	R	R	C	C	C
JM1	G	G	G	N	N	N	M	M	M	S	S	S	C	C	C	R	R	R
KC1	G	G	G	M	M	M	N	N	N	S	S	S	C	C	C	R	R	R
KC3	M	M	M	G	G	G	R	R	R	N	N	N	S	S	S	C	C	C
MC1	C	C	M	M	M	G	R	R	R	G	G	N	N	N	S	S	S	C
MC2	M	M	M	R	R	R	S	S	S	G	G	G	C	C	C	N	N	N
MW1	G	G	G	M	M	M	N	N	N	S	S	S	R	R	R	C	C	C
PC1	G	G	G	M	M	M	S	S	S	C	C	C	N	N	N	R	R	R
PC2	G	G	G	M	M	M	N	N	N	S	S	S	C	C	C	R	R	R
PC3	G	M	M	M	G	G	S	S	N	C	C	S	R	R	C	N	N	R
PC4	G	G	G	M	M	M	C	C	S	S	S	C	R	R	N	N	N	R
PC5	G	G	G	N	N	N	C	C	C	M	M	R	S	S	M	R	R	S
UIMS	S	M	N	R	N	M	M	S	S	N	R	R	G	G	C	C	C	G
QUES	N	N	N	M	M	S	G	G	R	R	R	C	S	S	M	C	C	G

## Threat to validity

Limitations encountered during this study are listed below:

1. The results obtained in this study are based on NASA, Li and Henry datasets which were developed using C, C++ , and Java. This study’s model is applicable to those paradigms only. Further research on various languages can be conducted to improve usability.
2. The code metrics considered for this study can be expanded with new code metrics that influence software code and design. It can play a significant role in predicting maintainability.
3. The NASA datasets are from automated satellite applications, but the use of real-time advanced automated application data will improve the SPM's reliability^34^.
4. The explainability of the predictions results still remains a concern.
5. The GA algorithm takes a huge amount of processing time and computational resource to produce the prediction results compared to other SMP models.

## Conclusion

This paper focused on removing the ambiguity in maintainability prediction models for predicting the maintainability of heterogeneous software. In this concern, various popular publicly available datasets of heterogeneous applications are considered and maintainability is predicted using GA over five popular techniques, namely, step-wise regression, support vector machine, neural networks, multivariate adaptive regression splines, and classification and Regression Tree. To choose the optimum model for predicting maintainability of heterogeneous software, multiple criteria decision-making model named TOPSIS is considered. The overall analysis has shown the efficiency of the proposed model over other popular maintainability prediction models. A range of possible future works have been identified while doing this research. There is a need of adapting real-time heterogeneous data for predicting maintainability. In addition, many other techniques and code metrics should be further adapted to enhance the estimation of maintainability.

## Data Availability

The datasets UIMS and QUES analyzed during the current study are available in the Li et al.^19^. The datasets JM1, KC1, KC3, MC1, MC2, MW1, PC1, PC2, PC3, PC4, and PC5 analyzed during the current study are available in the [Promise] and [Mendeley Data] repositories, [http://promise.site.uottawa.ca/SERepository/datasets-page.html], [https://data.mendeley.com/datasets/923xvkk5mm/1].

## Abbreviations

AI: Artificial intelligence
ANN: Artificial neural networks
BM: Base model
C&K: Chidamber & Kemerer
CART: Classification and regression tree
CKJM: Chidamber and Kemerer Java metrics
CPMP: Cross-project maintainability prediction
FLANN: Functional link artificial neural network
GA: Genetic algorithm optimized random forest
LOC: Line of code
MAE: Mean absolute error
MARS: Multivariate adaptive regression splines
MCDA: Multiple criteria decision analysis
MI: Maintainability index
NB: Naive Bayes
NN: Neural network
O and H: Oman and Hagemeister model
OO: Object-oriented
PCC: Pearson correlation coefficient
R^2^: R square
RMSE: Random mean square error
SLOC: Source line of code
SMP: Software maintainability prediction
SVM: Support vector machine
SWR: Step wise regression
